# Plague in Zimbabwe from 1974 to 2018: A review article

**DOI:** 10.1371/journal.pntd.0007761

**Published:** 2019-11-21

**Authors:** Amon Munyenyiwa, Moses Zimba, Tamuka Nhiwatiwa, Maxwell Barson

**Affiliations:** 1 Department of Biological Sciences, University of Zimbabwe, Mt. Pleasant, Harare, Zimbabwe; 2 University of Zimbabwe Lake Kariba Research Station, Kariba, Zimbabwe; University of California Davis, UNITED STATES

## Abstract

Plague is a zoonotic disease caused by the bacterium *Yersinia pestis* and is transmitted through the bites of infected rodent fleas. Plague is well known for causing 3 major human pandemics that have killed millions of people since 541 A.D. The aim of this Review is to provide an overview of the epidemiology and ecology of plague in Zimbabwe with special emphasis on its introduction, its potential reservoirs and vectors, and possible causes of its persistence and cyclic outbreaks. To achieve this, we carried out a search and document reported plague outbreaks in Zimbabwe. In the country, human plague cases have been reported in Hwange, Nkayi, and Lupane since 1974. The highest number of cases occurred in 1994 in the Nkayi district of Matabeleland North Province with a total of 329 confirmed human cases and 28 deaths. Plague is encountered in 2 different foci in the country, sylvatic and rural. Risk factors for contracting plague in the country include man-to-rodent contact, cultivation, hunting, cattle herding, handling of infected materials, camping in forests, and anthropic invasion of new areas. Plague is now enzootic in Zimbabwe, and the most recent case was reported in 2012, hence its effective control requires up-to-date information on the epidemiology and ecology of the disease. This can be achieved through continuous monitoring and awareness programs in plague-prone areas.

## Introduction

Plague is a reemerging flea-borne, rodent-associated zoonotic disease caused by the bacterium *Y*. *pestis* [1]. Plague epidemics have occurred in Asia, Africa, and South America, but most cases since the 1990s have occurred in Africa [1]. Plague was once widespread in Europe, but it has not been seen there for several decades [2]. The disease is characterized by long dormant periods that are punctuated by rapidly spreading epidemics and epizootics [3]. Currently, plague is one of the most important reemerging bacterial zoonoses in the world [4]. It is reemerging in countries where the disease was thought to have been eradicated, prompting the World Health Organization (WHO) to identify plague as an emerging disease [4, 5, 6]. The disease is notorious for causing 3 major human pandemics (Justinian Plague, Black Death, and The Oriental Plague) that killed millions from the 6th to the 19th centuries [7, 8]. At present, plague is one of only few infectious diseases subject to International Health Regulations (IHRs), which stipulate that any reported case of human plague be investigated and reported through appropriate authorities to WHO [4]. The causative bacterium has the potential to be developed as a biological weapon by terrorists [9]. In Zimbabwe, no cases of bioterrorism have been reported to date; however, given the current economic and political situation in the country, chances for developing *Y*. *pestis* as an agent for bioterrorism are likely to increase.

Plague is primarily a disease of wild rodents. The causative bacterium is transmitted to humans and other mammals mainly by dominant flea vectors such as *Xenopsylla cheopis*. Plague transmission occurs after the flea leaves the infected host, when the latter’s body temperature falls following its death either in the house or in the bush, due to disease or other causes, including hunting, predators, or poisoning as is the case of rodent control interventions at home [8]. Transportation of dead rodents or other plague reservoirs by hunters—such as in hunter’s pockets, on the shoulders, or in a carnivore’s mouth—facilitate the jumping or crossing over of fleas to humans and other mammals in domestic environments. In Zambia, pigs and goats were found to be infected with plague, and slaughtering the infected animals may be a risk factor for contracting plague in the country [8].

The disease is endemic in Southern, Northern, Eastern, and Central Africa, and plague cases have been recorded in these regions from the 19th to the 20th centuries [10]. African countries affected include Libya, Algeria, Tanzania, Uganda, Kenya, Senegal, South Africa, Zambia, Zimbabwe, Mozambique, Democratic Republic of Congo, and Madagascar [8]. Plague remains an epidemiological threat and a disease of major public health importance in Africa with the most reported cases in the continent [11]. Recent studies have indicated that Africa accounts for more than 90% of all human plague cases reported worldwide [12]. In Algeria, plague re-emerged in 2003 after 50 years of quiescence [13]. During the aforementioned year, WHO compiled and reported 2,651 plague cases and 175 deaths globally [11, 13]. Of all these plague cases and deaths, 80% were from 12 African countries [11]. Tanzania and Madagascar have recorded severe, widespread, and the most recent epidemics of the disease [14, 15, 16, 17], with Madagascar reporting 2,417 cases and 209 deaths in 2017 [17]. After this outbreak, 9 countries and overseas territories have been identified as priority countries in the African region for preparedness and readiness to plague outbreaks due to their air and shipping contacts with the island. These countries and overseas territories include South Africa, Mozambique, Tanzania, Seychelles, Comoros, La Reunion (France), Ethiopia, Mauritius, and Kenya [17]. Although the warning has not been extended to Zimbabwe, the high mobility of Zimbabweans because of economic hardships also puts the country at risk.

Continuous poverty at the household, community, and national levels; inequalities within and between sectors; and global climate change contribute to the perpetuation and reemergence of neglected tropical or zoonotic diseases such as schistosomiasis, anthrax, taeniasis, bovine tuberculosis, Rift Valley fever, and plague [18]. Here, we review human plague cases in Zimbabwe and risk factors associated with the epidemiology and ecology of the disease.

## Ecology and epidemiology of plague: An overview

### The *Y*. *pestis* bacterium

*Y*. *pestis* is a gram-negative, coccobacillus, nonmotile, non–spore-forming, and nonacid fast bacterium that belongs to the family Enterobacteriaceae. It falls under a group of bacilli that have low resistance to hostile environmental factors. The expression of metabolic pathways and virulence factors involved in *Y*. *pestis* host–pathogen interactions are regulated by environmental factors such as temperature, calcium concentration, and availability of iron [19]. Its optimum growth temperature is 28°C; if nutrients are available, it grows at temperatures ranging from −2°C to 45°C [20]. Inside the host where nutrients are available, its optimal growth temperature is 37°C, the body temperature of mammals. The plague bacterium grows optimally at 60% or higher relative humidity. Thus, temperature and moisture are critical for the persistence of *Y*. *pestis* and its transmission in plague-endemic areas.

In order to survive inside of the host and maintain a continual infection, *Y*. *pestis* developed a variety of mechanisms to evade and overcome the host immune system, especially the innate immune system [21]. In the case of bubonic, the disease is manifested as an acute, intransigent, and lethal infection, because the etiologic agent requires the death of its host in order to ensure perpetuation via transfer by the disenfranchised vector [22]. This strategy is dependent upon mounting an immediate overwhelming attack on the host before its immune system becomes capable of providing a significant defense, with a typical incubation period of 1 to 3 days in the case of pneumonic plague and 2 to 6 days for bubonic plague [21].This makes the disease difficult to treat, and by the time individuals are symptomatic, they are often close to death.

The virulent factors of *Y*. *pestis* depend on three plasmids, namely, Murine toxin (pMT)/Fraction (pFra); Calcium dependents (pCD); and Plasminogen activator gene, coagulase and pesticin (pPCP) [23]. The Plasminogen activator gene (pla) products are responsible for preventing the host blood from clotting there by facilitating the spread of *Y*. *pestis* in the body [24]. Murine toxin (MT), *Yersinia* outer membrane protein (Yop), and the structural gene for Fraction 1 (Fra 1) protein capsule offer resistance to bacterium from being engulfed by the monocytes or macrophages. MT is also essential for protecting the bacterium from being digested by the enzymes in the gut of the flea, thus enabling it to colonize the flea’s midgut and increase its transmission to the host [23].

*Y*. *pestis* is subdivided into 3 biotypes (antiqua, orientalis, and medievalis) depending on the ability to convert nitrate to nitrite and the ability to ferment glycerol [24]. *Y*. *pestis* var. *orientalis* does not ferment glycerol but reduces nitrate to nitrite, *Y*. *pestis* var. *antiqua* ferments glycerol and reduces nitrate to nitrite, and *Y*. *pestis* var. *medievalis* ferments glycerol but does not reduce nitrate to nitrite.

### The disease

The common form of plague is called bubonic and is caused by the bite of infected fleas. The plague bacillus penetrates at the bite and travels through the lymphatic system to the nearest lymph node, where it replicates itself. The lymph node then becomes tense, inflamed, and painful and is called a bubo. Septicemia plague develops when the infection is not promptly treated and cannot be localized by the regional lymphatic tissue resulting in its spread via the bloodstream, affecting various organs, including the spleen and lungs [25]. The resultant septicemia may be overwhelming and very rapidly fatal. Pneumonic plague results from the inhalation of plague bacilli from other pneumonic plague patients. The origin of pneumonic plague is a case of bubonic plague that develops into pneumonic plague. The latter is a new epidemiological cycle that eliminates the necessity for infected rodents and fleas as a source of transmission because it becomes transmitted on a purely man-to-man basis by droplet spread [25]. During the 2017 Madagascar plague outbreak, 77% of the cases reported were pneumonic, whereas 15% were bubonic [17]. The bubonic form of plague constitutes 90% of global cases, and more than 40% of the cases of primary bubonic plague progress to a secondary pneumonic plague, which is the deadliest form of the disease [4, 11]. The mortality rate of 70% or more has been reported in some African countries, due to a lack of availability of treatment in time, especially in remote rural areas, and the low density of health structures [11]. Susceptible rodents die from the disease, while some resist and become reservoirs of plague [25, 26, 27].

### Fleas as vectors of plague: Transmission of plague

The primary vectors of the plague bacterium are arthropods in the order Siphonaptera, commonly known as the flea [28]. Fleas that are associated with rodents, dogs, cats, and small mammals are considered important for maintenance and transmission of the plague bacterium [8, 10]. The disease is considered mainly to be transmitted by oriental rat fleas, *X*. *cheopis* (Rothschild) and *X*. *brasiliensis* (Baker), from infected rodents during epizootic and enzootic periods [29]. These flea species are known to transmit *Y*. *pestis* from infected animals to other warm-blooded animals through biting. Other flea species that may pose a risk of transmitting the plague bacterium are *Echidnophaga* spp., *Pulex irritans* (L.), *Ctenocephalides canis* (Curtis), and *C*. *felis* (Bouche) [30]. These flea species were considered to be potential vectors of the disease in Madagascar, where continuous outbreaks of the disease have been reported [16]. In Zimbabwe, the flea vector is usually from the genus *Xenopsylla*, of which *X*. *philoxera* is found in *Tatera* in desert areas while *X*. *brasiliensis* increases in numbers nearer the domestic environment and is associated with peridomestic and domestic rodents [31]. The 4 major rodent species—*T*. *leucogaster*, *Rattus rattus*, *Rhabdomys pumilio*, and *Mastomys natalensis*—are all hosts of *X*. *brasiliensis*, which is a known vector of the disease in Zimbabwe [32]. Zimba and colleagues reported an increased abundance of *Dinopyluss lypusus* and *C*. *calceatus* in Harare (Zimbabwe) and suggested that their roles in the transmission of plague in Zimbabwe needed further investigation [33]. In Tanzania, where several cases of plague have been reported, the rat fleas, especially *X*. *cheopis*, *X*. *brasiliensis*, and *D*. *lypusus*, are believed to play crucial roles in plague epizootics and epidemics due to the fact that they commonly infest susceptible hosts, which are usually abundant in plague foci [14,15].

### The role of mammalian reservoir hosts and rats’ susceptibility to plague

**Fig 1:**
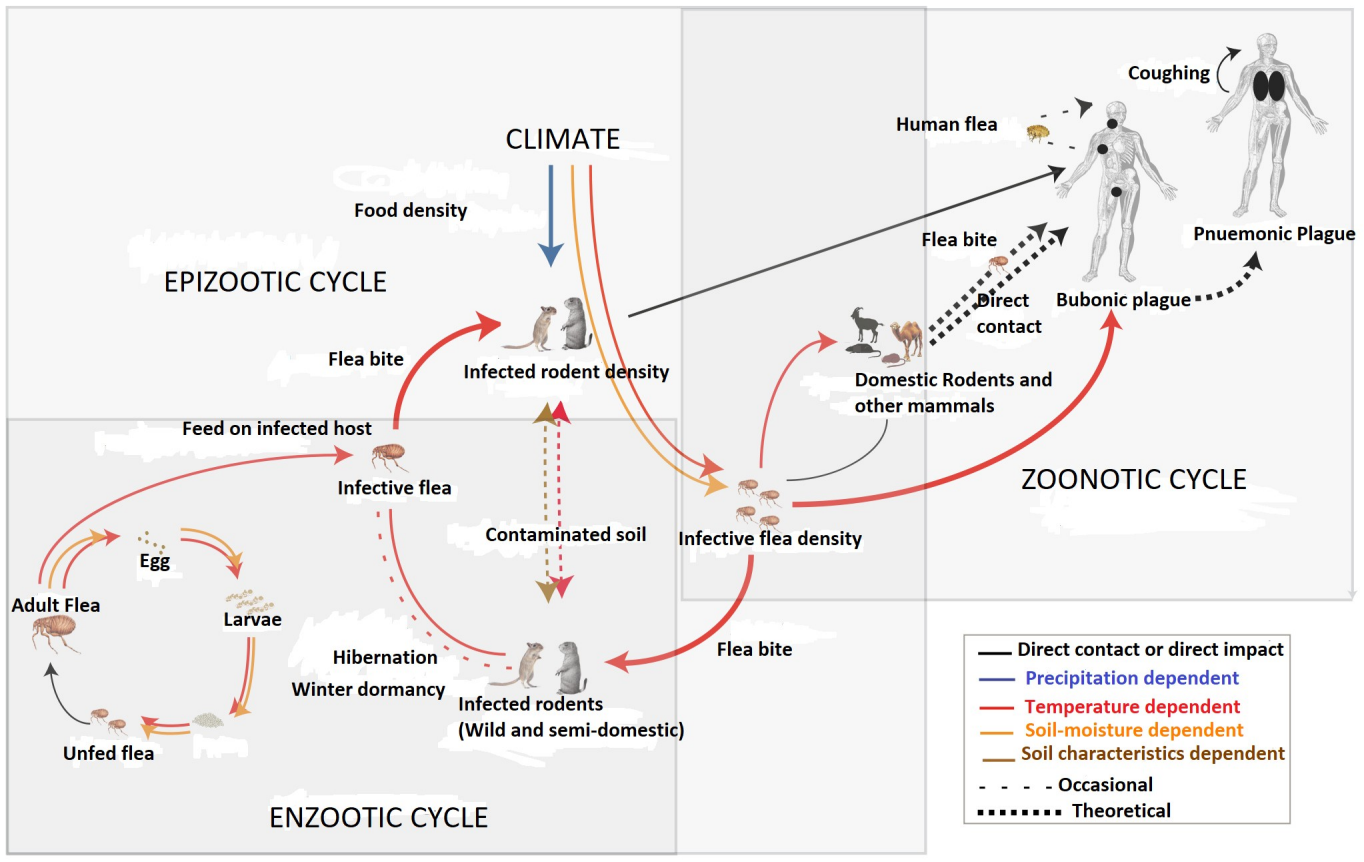
Transmission cycle of *Y*. *pestis* in a plague-endemic community [37]. Under favourable environmental conditions, populations of rodent species that are very susceptible to plague infection (*T*. *leucogaster* and *Mastomys coucha*) increase to high levels [38]. If these population increases occur in an area where there is a quiescent plague focus, the plague may break out in the susceptible rodent population. In plague-endemic areas, this population increase is crucial in plague transmission because a large number of mice and rats correspond to a large number of fleas [38]. Plague kills the susceptible rodents, and their infected fleas leave the carcass and seek new hosts, thereby spreading the infection rapidly throughout areas of high population.

Plague occurs naturally in wild rodents, which act as reservoirs of the bacteria [34]. Selected species of rats, mice, prairie dogs, voles, squirrels, cats, and dogs are suspected to be reservoirs of infection [35]. In Zambia, pigs and goats were also found to be reservoirs of the bacterium [8]. About 200 species of small mammals worldwide have been connected with the epidemiology of plague [36]. The animal hosts of plague are classified as enzootic or endemic (maintenance) hosts and epizootic or epidemic (amplification) hosts [4]. In several parts of the world, the bacterium is maintained in an enzootic cycle involving animal reservoirs and flea vectors (Fig 1). In enzootic hosts, mortality from plague infection is low although antibody surveys of the field population may show a positivity rate of up to 100% [4]. In an epizootic situation, plague organisms are occasionally introduced into rodent populations or areas of more susceptible host species.

### Persistence of plague in the soil

*Y*. *pestis* cannot be recovered from fleas, rodents, or any other hosts during inter-epizootic periods [39]. Persistence of *Y*. *pestis* in the soil was first postulated in Madagascar and Iran [40, 41]. The theory is that rodents become infected when they burrow in contaminated soil either via inhalation or ingestion, thereby restarting a new plague cycle. Though the exact mechanism remains unclear, scientific studies have established that *Y*. *pestis* can survive in the soil for at least 24 days under natural conditions [29]. This was previously established in 1963 by the inoculation of guinea pigs with soil samples collected from burrows, containing remains of *Meriones vinogradovi* that had been dead from *Y*. *pestis* infection for 7 to11 months [43]. Although *Y*. *pestis* may remain viable and virulent in soil, recent studies have demonstrated that the transmission cycle by exposure of susceptible mice to *Y*. *pestis–*contaminated soil seems unlikely under natural conditions [39]. This was because the infectious period was short lived and the transmission efficiency was also very low [42].

### Factors involved in plague dynamics

Factors involved in plague dynamics are landscape (elevation and vegetation), climatic variables [7, 43], or type of host [44]. The landscape may have an impact on the distribution of infectious diseases by influencing the population density and dispersal of vectors and hosts. Yersiniosis infection is a highly virulent, reemerging disease, the ecology of which has been scarcely studied in Africa [45]. Human seroprevalence data for the major plague focus of Madagascar suggest that plague spreads heterogeneously across the landscape as a function of the topographic relief [39].

In Central Asia, *Y*. *pestis* persistence and dynamics were often named according to the dominant host species in that locality [46]. Understanding of host–vector relationships is important to identify the role of arthropod vectors as well as mammalian reservoirs in the maintenance of various diseases [47]. Factors such as precipitation and temperature may also affect the distribution and abundance of key hosts and vectors involved in *Y*. *pestis* transmission [48]. Anthropogenic activities are also involved in altering plague dynamics due to modification of landscape and faunal composition of the foci and adjacent areas, thereby increasing human cases. In the current transitional state as a whole, plague is at risk of becoming a public health problem in poor countries in which ecosystem erosion, anthropic invasion of new areas, and climate change increase human contact with rodent reservoir systems [49].

## Plague in Southern Africa

Plague invaded Southern Africa for the first time during the last great pandemic, which began its devastating, worldwide spread from Hong Kong in 1894 [31]. In 1914, sporadic cases of plague occurred in remote rural areas of the eastern Cape Province, the northern Orange Free State, and the southwestern Transvaal of South Africa. Since then, it was eventually demonstrated that wild rodents had become infected with *Y*. *pestis* and that they were forming a plague reservoir from which humans may get infected from time to time [50]. The distribution of human plague in Southern Africa is linked to the distribution of the multimammate mouse, *M*. *natalensis* [51]. In Southern Africa, the peridomestic rat, *M*. *natalensis*, acts as the link between feral rodents and the human environment where *Rattus* may or may not be involved in plague transmission [52]. The gerbils *T*. *brantsii*, *T*. *leucogaster*, and *T*. *afra* play an important role in Southern African plague epidemiology [4]. In South Africa, studies have been done to determine whether the sibling species of rodents—*Aethomys chrysophilus*, *T*. *leucogaster*, *M*. *natalensis* (*M*. *coucha*), and *A*. *namaquensis*—differed in their potential roles as reservoirs of plague. *A*. *namaquensis* was found to be more plague sensitive than *A*. *chrysophilus*, and the two may play different roles in the plague cycle [51].

### Plague in South Africa

Human plague occurred in 1982 after a quiescent period of 10 years; a total of 19 cases and 1 death were reported in Sothern Cape of Good Hope Province in a Village near Port Elizabeth [4]. During the outbreak, the plague antibody was found in 2 rodent species—the vlei rat, *Otomys irroratus*, and the four-striped mouse, *R*. *pumilio* [4]. Serological surveys were further contacted in 1982, and sera from 3,012 rodents of the 24 species trapped were tested for the presence of the antibody to the Fra 1 antigen of *Y*. *pestis* by passive haemagglutination [51]. Of the 24 species investigated, antibodies were found in 7 (0.23%) rodents of 3 species, *Desmodillus auricularis* and *T*. *brantsii* in the Northern Cape Province and *R*. *pumilio* in the eastern Cape Province [51].

### Plague in Zambia

Zambia recorded 320 cases from 1987 to 1997 [4]. Zambia experienced plague outbreaks in 3 zones, the eastern, southern and northwestern regions of the country [8, 53]. Factors that contributed to plague outbreaks and spread in the country included heavy rains, which were usually followed by a large increase in rodent and flea populations, sociocultural human behaviour, and community lifestyle [53].

### Plague in Mozambique

Mozambique reported 124 cases of plague from 1976 to 1978 in Tete Province [54]. There was a drought in the country during this period, and it is possible that plague was transmitted to domestic rodents (*Rattus*) by peridomestic rodents, particularly *M*. *natalensis* [4]. Man-to-rodent contact was also implicated in plague transmission. Women and children were reported to use sacks to collect rodents that were then killed, skinned, and dressed before consumption [4]. In 1994, plague reemerged after 15 years of quiescence in the Mutara district of Tete Province, and 216 cases and 3 deaths were reported [55, 56]. In 1997, human plague cases were also reported in the same area, 825 cases and 18 deaths [57].

### Plague in Botswana

In Botswana, an outbreak of plague occurred from 1989 to 1990 [58]. An outbreak began in the Boteti district that lasted 24 weeks; 173 cases and 12 deaths were reported [58]. After a bumper harvest of 1989 that resulted in increased rodent population and their infective fleas, a large epizootic of plague among wild rodents penetrated the human environment and caused an epizootic among domestic rats, which then transmitted plague to humans [4, 59].

### Plague in Angola

Human plague occurred in 1980 to 1981 at Bocoio in the Province of Benguela, and 27 cases (4 deaths) were recorded [60]. This was the second outbreak after the first outbreak in 1975 [61].

### Plague in Malawi

In 1994, 4 confirmed bubonic plague cases occurred in the Nsanje district among Mozambican refugees living in the Mankhowe refugee camp and surrounding areas [56]. Another human outbreak occurred in Chikwawa, Nsanje, and Ntchisi districts in 1997 with a total of 582 cases and 11 deaths [56].

### Plague in Namibia

In Namibia, human plague occurred between 1954 and 1955 with a total of 8 cases and 2 deaths [4]. Other plague cases were reported from 1960 to 1962 (92 cases and 10 deaths), from 1967 to 1969 (113 cases and 49 deaths), from 1972 to 1975 (16 cases and 10 deaths), and in 1974 (102 cases and 5 deaths) [4].

## Materials and methods

The literature review was conducted using the online databases PubMed and HINARI. Hard-copy textbooks with information relevant to plague were obtained from the University of Zimbabwe library. A thorough search of the work was then undertaken in Zimbabwe, for 1974 to recent findings. The work includes published reports from WHO and the Ministry of Health and Child Care, with particular emphasis on the epidemiology of plague in Zimbabwe. A systematic search strategy was employed using the following search terms: ‘Africa’ or the name of any African country (e.g., ‘Rhodesia or Zimbabwe) and ‘plague’ (e.g., ‘*Yersinia pestis*’). The search was refined to manuscripts that reported the occurrence of animal or human plague in Southern, Central, Eastern, and Northern Africa and the whole world. The DOI for this protocol can be found at https://dx.doi.org/10.17504/protocols.io.3vvgn66.

## Results

### Plague in Zimbabwe

#### The affected area

Three plague epidemic-prone areas in Zimbabwe are Hwange (18.3559° S, 26.5020° E), Nkayi (18.992° S, 28.9005° E), and Lupane (18.9300° S, 27.7593° E) [18]. The Nkayi and Lupane districts are semi-arid and are made up of largely savannah forests where cattle are grazed. The forests act as suitable habitats for rodents and other small mammals that may play important roles as potential reservoirs of the plague bacterium. Most of the people in the Nkayi and Lupane districts are peasant farmers relying on small-scale production of maize and sorghum and rearing of cattle [62, 63].

Hwange National Park covers 14,651 square kilometres and is Zimbabwe’s largest and oldest national park. It is situated in the northwest of the country. Its western boundary, which is unfenced, is bordered by Botswana along its length. Hwange National Park is an area of mixed veld consisting of teak woodlands and mopane savanna woodland on the Kalahari Sands in the south. The *Acacia* sp. tree savanna is in the centre of the National Park, whereas the mopane Savanna woodland is to the north. Vleis and grasslands are also present. Most of the Zimbabwean game species are represented among the mammal populations, including members of the order Rodentia and Lagomorpha, which have proven to be susceptible to *Y*. *pestis* [64].

#### Plague in Zimbabwe (1974–1975)

The first incidence of plague in Zimbabwe occurred in September 1974 in Hwange National Park, and it was fully reported [66] (Table 1). The first case was reported on the September 25, 1974, when P. N., a 23-year-old male, was admitted to the hospital in Hwange with a history of an injury to his right shoulder [64]. The patient was examined for any symptom of injury clinically or radiologically. No sign of injury was observed. The following day, the patient developed pyrexia and cellulitis of the right pectoral region and upper part of his arm. Penicillin therapy was administered to the patient, but unfortunately, the patient collapsed suddenly and died in the early hours of the morning on September 27, 1974.

**Table 1:** Recorded human plague cases from 1974 to 2018 in Hwange, Nkayi, and Lupane, Matabeleland North Province, Zimbabwe.

Number	Year	District	No. of recorded cases	No. of recorded deaths	Reference
1	1974	Hwange	23	8	[4]
2	1975	Hwange	34	12	[4]
3	1982	Lupane	3	2	[4]
4	1983	Lupane	1	0	[4]
5	1985	Lupane	1	1	[4]
6	1994	Nkayi	329	28	[62]
7	1997	Nkayi	8	2	[4]
8	1998	*	8	2	[4]
9	1999	*	9	2	[4]
10	2012	*	1	0	[65]

*Location not available.

On September 26, 1974, P. N.’s brother R. N., aged 16, was also found dead in the house in which they stayed together, and his body was brought to Hwange Hospital. Their bodies were sent for post-mortem examination to Bulawayo’s Mpilo Hospital. The most valuable findings at P. N.’s autopsy were swelling of the right upper limb, axilla, and pectoral area [64]. Oedema was observed in the pectoral muscles and subcutaneous tissues of the patient when the body was dissected. This was the only useful diagnostic feature observed. The fluid was slightly mucoid and serosanguinous. Gross lymphadenopathy was not observed on R. N.’s autopsy, but all organs were congested, and the body showed a fair degree of autolysis; it was compatible within 5 hours in a metal casket on top of a Land Rover while being transported from Hwange to Bulawayo. R. N.’s autopsy revealed haemorrhagic areas in the scalp, left chest, neck, and retroperitoneal areas. Some decomposition had occurred, and the cause of death was difficult to ascertain. From both bodies, samples of blood, spleen, and oedema fluid were taken for bacteriological and serological examinations. An immediate microscopic examination was made of direct smears, and all samples were cultured. Initial cultures were made onto blood agar for aerobic and anaerobic growth and onto MacConkey incubated at 36°C [64]. Haemorrhagic fluid from the connective tissue and spleen of R. N. exhibited gram-negative, bipolar-staining bacteria consistent with *Y*. *pestis*. The samples were later confirmed by culture and animal inoculation in both cases. Human plague was diagnosed in Zimbabwe for the first time in this manner. On October 27, just 5 weeks after the second case, A. N., a 12-year-old boy, was admitted to a mission hospital in Lupane district, gravely ill with a raised body temperature of 39°C and enlarged, tender cervical and inguinal glands. Bubonic plague was again suspected, and antibiotics were administered to the patient. The patient responded very slowly to penicillin, tetracycline, and streptomycin [66, 64]. The patient was traced, and it was found that he came from an isolated village in a forest area where rodent burrows were abundant, and his family members had observed more rodents than usual in the area [64].

The intention is not to describe individual cases of plague that occurred in Zimbabwe, but some of the cases are worthy of description. Note that during the outbreak, patients with moderate pyrexia and lymphadenitis did not appear unduly ill on admission, but they collapsed and died unexpectedly some hours later. Inguinal lymphadenitis (“imbilapi” in local language) was a common condition in barefooted children in areas where human plague had been reported in the country, and parents know that it disappears when the first lesions heal. There is a serious danger that cases of plague in rural areas may not be recognized as serious illness and patients may not be brought in for treatment in time. The buboes may also not be so conspicuous and may even be missed in mild cases. Due to the unspecific nature of these symptoms, isolated cases may not be recognized and just be recorded as “pyrexia of unknown origin” (PUO) [63]. Another 2 patients were deeply jaundiced on admission to rural hospitals, and 2 developed severe anaemia critical enough to warrant blood transfusion [66]. Fortunately, the clinicians concerned have developed a high index of suspicion regarding plague, and the correct diagnosis was made. There are higher chances that plague can be misdiagnosed as anaemia or any other disease with similar symptoms, therefore clinicians in plague-endemic areas must be aware of the disease. The health system was also slow in diagnosing the infection, and this in turn increases mortality rates during outbreaks.

After plague had been diagnosed for the first time in Hwange National Park, more cases of human plague occurred in different parts of the Nkayi and Lupane districts. Twenty-three cases and 8 deaths were reported in 1974 followed by 34 cases and 12 deaths in 1975 [4]. Taylor and colleagues reported a total of 97 confirmed human plague cases from 1974 to 1975 in Zimbabwe [67]. The distribution of confirmed human cases during the1974–1975 outbreak was restricted to the Shangani River system [67]. The distribution of human cases was much more restricted than the distribution of the actual epizootic as evidenced by the wide presence of plague antibodies in dogs around the country [67]. A plague epizootic was also reported in 1977 and 1978 in the northern part of Zimbabwe, in Mashonaland Central Province [67]. The epizootic was without reported occurrence of human plague even though all the positive dogs live in close contact with owners in crowded villages [67].

During the 1974–1975 outbreak, dead rodents and squirrels were observed, and this served to raise suspicion of the occurrence of plague in the area [63]. The susceptible rodent species identified were the three-striped mice (*R*. *pumilio*), the multimammate mouse (*Mastomys* spp.), the local gerbil *T*. *leucogaster*, and the bush squirrels (*Paraxerus* spp.) [63, 64]. In order to establish the geographical extent of the 1974–1975 outbreak and the degree of plague endemicity in Zimbabwe, Cruikshank and colleagues and Taylor and colleagues contacted a nationwide survey [67, 68]. They investigated the presence of *Y*. *pestis* in the blood of dogs, small mammals, and humans. The survey showed that antibodies to *Y*. *pestis* in dog sera was above 5% positivity in the north and northwestern parts of the country, whereas in areas along the watershed and around Harare, infection rates of these animals were less than 5% [67]. According to Taylor and colleagues, results of serological tests in Zimbabwe from 1975 to 1978 showed the presence of antibodies to *Y*. *pestis* in 4.24% of 3,964 dog sera, 0.86% of 1,048 human sera, and 0.88% of 454 small mammal sera [67]. Findings of the 1974–1975 survey indicated that human sera were much less sensitive as indicators of plague than dogs. In small mammals, antibodies to plague were demonstrated in the following rodents: *A*. *chrysophilus*, *A*. *namaquensis*, *Peripatopsis capensis*, and the local gerbil *T*. *leucogaster* [67].

#### Plague in Zimbabwe (1981–1985)

Since the 1974–1975 epidemic, sporadic cases of human plague continued to occur in Matabeleland North Province in 1982, 1983, and 1985 (5 cases, 3 deaths) [4, 11]. In January 1982, two sisters aged 8 and 10 were admitted at St. Lukes Hospital, which is situated in the Lupane district of Matabeleland North Province, both patients with fever, patient A with a bubo in the left axilla, and patient B with an abscess and oedema on the right breast [63]. Both patients died the day they were admitted with septicaemia and evidence of lung congestion. A total of 5 human plague cases and 3 deaths were confirmed. Investigations were done to determine the possible sources of infection. Rodents captured during the investigations were all *T*. *leucogaster*; 20% of the rodents captured died, and *Y*. *pestis* was isolated from both animals [63]. The remaining rodent captures all showed higher antibody titres to *Y*. *pestis*. It was also noted that the houses were constructed in a virgin woodland near Tshongokwe mission (grid reference 27° 23 E, 18° 38 S), and in one of the cases, the family cat was known to regularly bring rats to the house [63]. Other sporadic cases of plague were reported in 1983 (1 case, 1 death) and in 1985 (1 case, 0 deaths) [4]. Unfortunately, all of these cases were not fully reported.

#### Plague in Zimbabwe (1994–2018)

In 1994, more cases of human plague than ever before were recorded in Zimbabwe. A total of 329 human plague cases and 28 deaths were reported [4, 62]. In mid-September 1994, district health officials in the Nkayi district, in the Matabeleland North Province of western Zimbabwe, were informed by the police of 5 unexplained deaths that had occurred in homes [62]. All were children who had died 4 weeks prior. All died within a few days of the onset of the illness. The illness was characterised by fever, headache, and localized tender lymphadenopathy. Bubonic plague was suspected, and the district had reported sporadic cases of the disease since 1974 [63]. The highest number of cases occurred in October to November during the rainy season; 80% of the patients were under 15 years old. During this period, many areas in Zimbabwe had reported an increase in rat populations [62]. After this outbreak, it appeared likely that further outbreaks would occur in the future, and there was still no clear idea of what might be the potential risk factors. A case-control study was conducted, and it was noted that factors significantly associated with being a plague case were herding cattle, being aged 10 years or older, hunting, and having a sick cat at home [62]. During the 1994 outbreak, school children were also reported to be in constant contact with rodents; they killed rodents and carried them in their pockets in order to scare the girls or feed their dogs so that the dogs would run fast during hunting [62].

Other plague cases in Zimbabwe occurred in 1997 (8 cases and 2 deaths), 1998 (8 cases and 2 deaths), 1999 (9 cases and 2 deaths), and 2012 (1 case and 0 deaths) [4, 65]. All of these other cases were not fully reported, and the reasons for this are unclear. No cases of human plague were reported from 2013 to 2018 in the country.

## Discussion

### Sylvatic plague, outbreak risk factors, and control in Zimbabwe

Plague in Zimbabwe is present in isolated foci among rodents that are relatively resistant to the infection. Usually, the sylvatic rodent reservoirs are species that are susceptible to the infection but resistant to the disease [4]. Due to the sylvatic nature of plague in Zimbabwe, its transmission to humans most likely involves a complex interaction among wild rodents, semi-domestic rodents (*Mastomys* species), domestic rodents (*Rattus* species), and their flea vectors [27, 67]. In Matabeleland North Province, humans mostly get infected after being bitten by infected fleas when they enter habitats of wild rodents through activities such as herding cattle, cultivation, and hunting [62, 63, 64, 68, 69]. Because plague is largely sylvatic in Zimbabwe, it is the rural peasant farmer population who are most at risk of infection. People in Nkayi and Lupane districts rely mainly on cattle rearing and subsistence farming for their basic income and food for their families [62]. These occupational activities expose them to the risk of plague infection. In Hwange National Park, game rangers and tourists who camp in the forests are most at risk of plague infection. Isolated human cases of plague, as recorded from 1981 to 1985, most likely resulted from flea bites in isolated pockets of active plague or from accidental infection due to handling infected animals [63].

Man-to-rodent contact is a risk factor for contracting plague in Zimbabwe. The results of a survey conducted after the 1994 outbreak in Matabeleland North Province showed that children were in constant contact with rodents [62]. School children in the Nkayi district reported that they killed rats to feed their dogs so that they would run fast when hunting. In the Tsholotsho district, schoolboys also reported that they keep dead rats in their pockets in order to scare the girls. Direct contact with infected materials is a well-known route for plague transmission [8]. People in Nkayi and Lupane districts tend to hunt and transport or dispose of dead rodents in a manner that facilitates infective fleas jumping from dead rodents to humans or any other domestic animal, thereby facilitating the transmission of the disease. Other risk factors for contracting plague in Zimbabwe include having a sick cat at home, being older than 10 years, and resettlement [62, 63]. People older than 10 years living in rural areas are associated with activities such as herding cattle, hunting, and farming. These occupational activities require people to venture into the bush, whereas the anthropic invasions of new areas increase contact of the human population with plague reservoirs systems in the country.

During the 1974–1975 outbreak, control of the disease was done through educating people about plague in rodents and its transmission to humans, quarantine, and chemoprophylaxis using sulphadimidine tablets at the dosage of 2 tablets (3 times a day for 5 days) [66]. Interestingly, flea control was done using fenitrothion due to the fear of DDT insecticide resistance. In order to improve knowledge of the control of the disease, Cruikshank and colleagues carried out a surveillance study on rodents and suggested that there was no evidence that plague was present in Zimbabwe either in man or in animals for any length of time prior to the clinical human outbreak in September (1974). They went on to propose that human cases may have followed quickly upon the invasion of the Hwange area with infected rodents and their associated fleas. *A*. *chrysophilus* tested positive for plague in Gweru during the survey, and it might have acted as a potential reservoir of the plague bacterium together with other rodent species [68].

### The complex relationship between epizootics and plague outbreaks in Zimbabwe

During plague outbreaks in Zimbabwe, dead rodents are usually observed, indicating that an epizootic occurred. The distribution of plague epizootics and human cases is complex in the country. During the 1974–1975 outbreak, the distribution of human cases was much more restricted to the Shangani River compared to the actual distribution of the disease in the zoonotic cycle [67]. Human cases were only reported in Matabeleland North Province. In contrast, serological surveys showed that plague is widespread in Zimbabwe as demonstrated by the presence of antibodies to *Y*. *pestis* among sampled dog populations, including areas around Harare [67, 68]. A plague epizootic was also reported in Mashonaland Central Province from 1977 to 1978, but no human cases were reported [68]. Plague is therefore widespread in rodents and dogs in the country, but interestingly, human cases have only been reported in Nkayi, Lupane, and Hwange. The reasons for this are unclear, and the actual relationship between human cases and the real distribution of plague in enzootic foci in the country is questionable. Davis and colleagues have demonstrated serological evidence of plague in areas where either it has never been reported or it has been absent for several years [70].

The rodent species *A*. *chrysophilus*, *A*. *namaquensis*, *T*. *leucogaster*, and *P*. *capensis* were all found to be infected with *Y*. *pestis* during epizootics, and they play different roles in plague epidemiology in the country [67]. In South Africa, the primary reservoir of the plague bacterium in the wild is the gerbil *T*. *brantsii* [52], whereas in Zimbabwe, rodents such as the rock rat, *A*. *chrysophilus*, and the multimammate mouse, *M*. *natalensis*, are relatively resistant to plague infection and are most likely to act as potential sylvatic reservoirs of *Y*. *pestis* [67].Susceptible rodents to plague infection in Zimbabwe are *T*. *leucogaster* and *M*. *coucha* [64]. These susceptible rodents play an important role in plague transmission in the country. Plague kills the susceptible rodents, and their infected fleas leave the carcasses and seek new hosts, thereby spreading the infection rapidly throughout the area [63].

In addition, the strain of *Y*. *pestis* isolated in Zimbabwe during the 1974 to 1975 outbreak does not ferment glycerol, but it reduced nitrate to nitrite [64]. Therefore, the strain of *Y*. *pestis* in Zimbabwe is of the orientalis type (*Y*. *pestis* var. *orientalis*), which was also reported in South Africa [64] and in Madagascar [39]. This strain spread all over the world during the third pandemic.

Studies conducted in Zimbabwe also revealed that humans are less sensitive as indicators of the presence of plague than dogs [68].These findings coincide well with those of William and colleagues in Navajo reservations (Arizona and New Mexico), which revealed that dogs are highly resistant but not refractory to plague and that testing of sera from domestic dogs for plague antibodies can provide useful information on the epidemiology of plague [71]. Future studies in Zimbabwe should focus on dog and rodent serology as the most sensitive way of delimiting plague foci that may need further investigations. Now that it has been established that plague is now enzootic in Zimbabwe and probably over a wide area [67, 68], monitoring should be carried out on a regular basis.

### Climate and plague in Zimbabwe

Climate has long been thought to be a key factor in the alternation between quiescent and active periods of human plague. The abundance of rodent fleas is affected by ambient temperatures, rainfall, and humidity, with warm moist weather providing a likely explanation for higher flea indices. This explains the increased number of plague cases during the rainy season (October and December) in the Nkayi district in 1994 [62]. Zimba and colleagues also recorded higher flea indices for the efficient vector of *Y*. *pestis*—*X*. *brasiliensis*—during the hot-wet season in Harare, the capital city of Zimbabwe [32]. A high Specific Flea Index increases the risk of plague transmission to humans in endemic areas. In Zambia, most cases of plague were also reported during the rainy season [8]. This period is characterized by warm moist conditions that are favourable for the development of flea vectors in Southern Africa. In Madagascar, where almost one-third of human plague cases reported worldwide occur, the El Niño Southern Oscillation (ENSO) and Indian Ocean Dipole (IOD) events, together with the corresponding increase in temperature and rainfall, show strong associations with an increase in plague incidence [72]. The increased moisture associated with high rainfall supports the survival and reproduction of fleas [37, 72].

## Conclusion

The Review highlights the complexity of plague epidemiology and ecology in Zimbabwe. Plague is sylvatic in nature in Zimbabwe. Humans get infected when they visit zones of active plague foci through activities such as cultivation, herding cattle, and hunting. Resettlement or anthropic invasion of news areas is also a risk factor for contracting plague in the country because it increases the contact of the human population to reservoir systems. *Mastomys* spp. and their infective fleas act as the true link between the sylvatic plague cycles and the domestic plague cycles during epizootics. The distribution of human plague cases in Zimbabwe is much more restricted to Matabeleland North Province compared to the wide distribution of actual epizootics in the country. This raises more questions than answers. The rodent species *A*. *chrysophilus* and *M*. *natalensis* are relatively resistant to plague infection and most likely act as potential reservoirs of *Y*. *pestis* during quiescent periods. *T*. *leucogaster* and *M*. *coucha* are highly susceptible to plague and are known to be infested with *X*. *brasiliensis*, the principal plague vector in the country. Humans are not sensitive as indicators of plague in the country, therefore future studies on the ecology and epidemiology of plague should focus on dogs and rodents. Furthermore, there are higher chances that plague may not be recognized as a serious illness, therefore enhancing public health education in order to sensitize the communities is desirable. Plague is now enzootic in Zimbabwe, and the occurrence of other epidemics and epizootics in the future is inevitable. The pattern of human plague cases also indicates that plague occurrs every 5 to 8 years in the country. Hence, up-to-date information on the incidences and distribution of the disease is required in order to develop comprehensive control measures for the disease in the country. This can be achieved through a surveillance program that collects, analyses, and interprets clinical, epidemiological, and epizootiological data on plague.

**Box 1. Key learning points**

- Zimbabwe is among the African countries that have recorded human plague cases during the past 25 years, with the most recent case reported in 2012.
- In Zimbabwe, plague occurs mainly in rural areas and wildlife areas (Nkayi, Lupane, and Hwange National Park), and it is largely sylvatic in nature, involving endemic rodents and fleas.
- The dominant rodent species in Zimbabwe and plague-prone areas of Zimbabwe are *T*. *leucogaster*, *M*. *natalensis*, *R*. *rattus*, and *R*. *pumilio*; *X*. *brasiliensis* is regarded as the principal plague vector in or near domestic settings.
- The distribution of human plague in Southern African countries is linked to the distribution of species B of the *M*. *natalensis* (*sensu stricto*) species complex, and *A*. *chrysophilus* and *M*. *natalensis* are relatively resistant to plague infection and most likely act as potential sylvatic reservoirs of infection.
- Plague is now enzootic in Zimbabwe, and human cases have been reported since 1974; therefore, occurrence of other epidemics and epizootics in the future is inevitable considering the reemerging nature of the disease and that the disease is currently neglected in the country.

**Box 2. Top five papers**

1. Cruickshank JG, Gordon DH, Taylor P, Naim H. Distribution of plague in Rhodesia as demonstrated by serological methods. *Central African Journal of Medicine*. 1976; 22: 127–130.
2. Manungo P, Peterson DE, Todd CH, Mthamo N, Pazvakavambwa B. Risk Factors for Contracting Plague in Nkayi District, Zimbabwe, *Central African Journal of Tropical Medicine*. 1998; 44:173–175.
3. Pugh A O, Parker D A. Plague: Rhodesia's first recorded outbreak. *Central African Journal of Medicine*. 1975;21:93–96.
4. Taylor P, Gordon DH, Isaacson M. The Status of Plague in Zimbabwe. *Annals of Tropical Medicine and Parasitology*. 1981; 75:165–173.
5. WHO. “Plague manual. Epidemiology, distribution, surveillance and control,” *WHO/CDS/CRS/EDC/99*.2: 1999. pp.1–26. Available from: https://www.who.int/csr/resources/publications/plague/WHO_CDS_CSR_EDC_99_2_EN/en/ February 29, 2019. [cited 2019 Feb 29].
