# Correction: Plague in Zimbabwe from 1974 to 2018: A review article

**DOI:** 10.1371/journal.pntd.0008522

**Published:** 2020-07-10

**Authors:** Amon Munyenyiwa, Moses Zimba, Tamuka Nhiwatiwa, Maxwell Barson

After this article [1] was published, concerns were raised about attribution of some included content.

Fig 1 in [1] is reused from [2], which was published under the CC0 public domain dedication. While the figure title in [1] included a citation to the *PLOS Pathogens* article (reference 37), the legend did not explain clearly that the figure was reused from the other source. The authors apologize for this issue and provide an updated Fig 1 legend, below.

In addition, some of the text in [1] overlaps with previously published work. This includes the following text excerpts for which we provide the relevant citations with this notice:

- The “Fleas as Vectors of Plague: Transmission of Plague” section includes text that overlaps with [3]: “The disease is considered…considered to be potential vectors of the disease.”
- The “Persistence of Plague in the Soil” section includes text that overlaps with [4]: “*Y*. *pestis* can survive in the soil … unlikely under natural conditions.”
- In the “Factors involved in Plague Dynamics” section, there is text overlapping with
  ○ [5]: “distribution of infectious disease…function of the topographic relief”
  ○ [6]: “affect the distribution and abundance… contact with rodent reservoir systems”
- In the “Plague in Southern Africa” section, the majority of text in the following excerpt overlaps with [7]: “The distribution of human plague in Southern Africa…roles in the plague cycle.”
- The first two sentences of the “Climate and plague in Zimbabwe” section overlap with [8].

**Fig 1:**
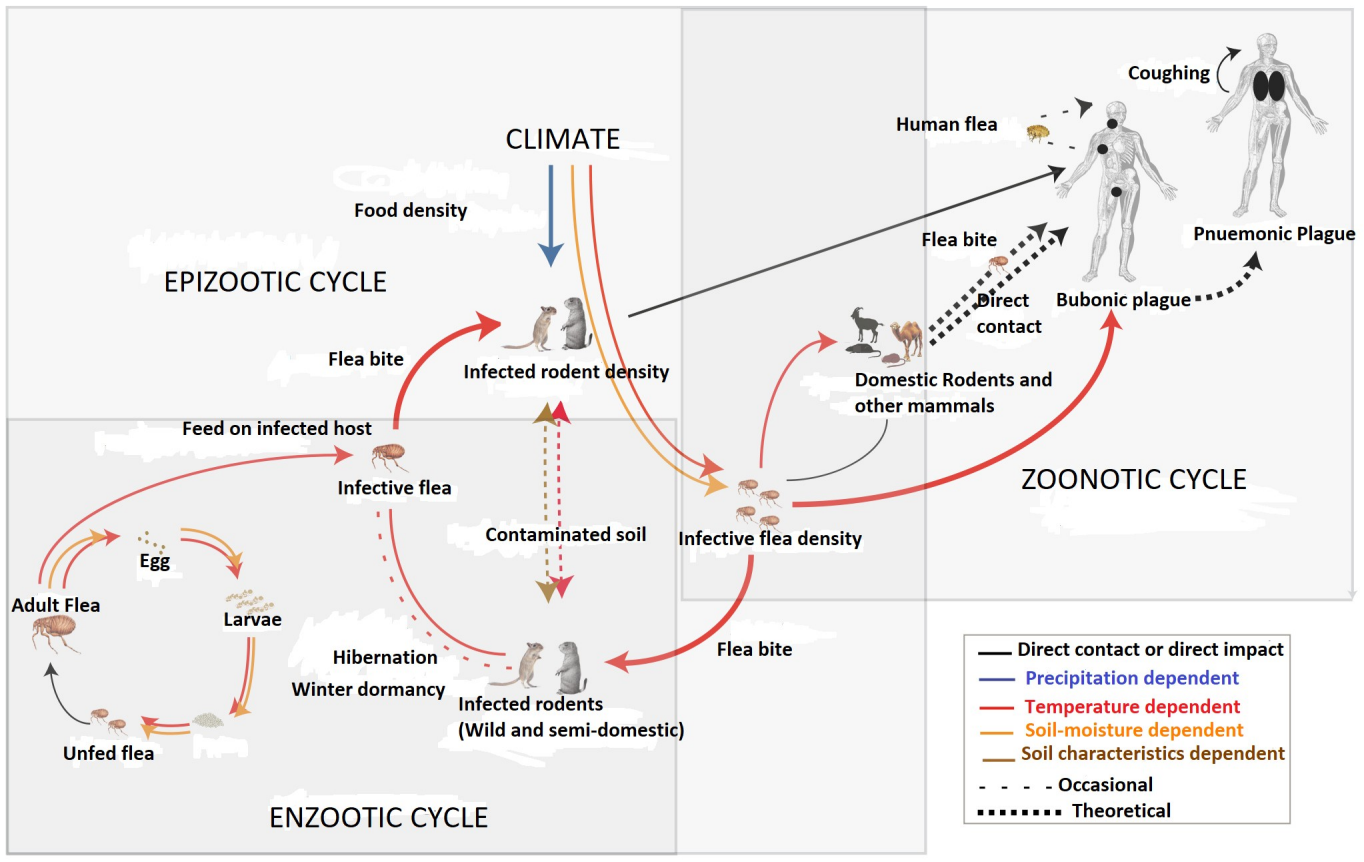
Transmission cycle of *Y*. *pestis* in a plague-endemic community. This figure was originally published by Ben Ari et al. [37] which was made available under the Creative Commons CC0 public domain dedication. Under favourable environmental conditions, populations of rodent species that are very susceptible to plague infection (*T*. *leucogaster* and *Mastomys coucha*) increase to high levels [38]. If these population increases occur in an area where there is a quiescent plague focus, the plague may break out in the susceptible rodent population. In plague-endemic areas, this population increase is crucial in plague transmission because a large number of mice and rats correspond to a large number of fleas [38]. Plague kills the susceptible rodents, and their infected fleas leave the carcass and seek new hosts, thereby spreading the infection rapidly throughout areas of high population.

## References

[1] Munyenyiwa A, Zimba M, Nhiwatiwa T, Barson M (2019) Plague in Zimbabwe from 1974 to 2018: A review article. PLoS Negl Trop Dis 13(11): e0007761 10.1371/journal.pntd.0007761 31751348 PMC6871778

[2] Ben Ari T, Neerinckx S, Gage KL, Kreppel K, Laudisoit A, Leirs H, et al (2011) Plague and Climate: Scales Matter. PLoS Pathog 7(9): e1002160 10.1371/journal.ppat.1002160 21949648 PMC3174245

[3] Nyirenda SS, Hang’ombe BM, Kilonzo BS, Kangwa HL, Mulenga E, Moonga L. (2017) Potential Roles of Pigs, Small Ruminants, Rodents, and Their Flea Vectors in Plague Epidemiology in Sinda District, Eastern Zambia. J. of Medical Entomology 54(3):719–725. 10.1093/jme/tjw220 28399281

[4] Andrianaivoarimanana V, Kreppel K, Elissa N, Duplantier J-M, Carniel E, Rajerison M, et al (2013) Understanding the Persistence of Plague Foci in Madagascar. PLoS Negl Trop Dis 7(11): e2382 10.1371/journal.pntd.0002382 24244760 PMC3820717

[5] Eisen RJ, Borchert JN, Mpanga JT, Atiku LA, MacMillan K, Boegler KA, et al (2012) Flea Diversity as an Element for Persistence of Plague Bacteria in an East African Plague Focus. PLoS ONE 7(4): e35598 10.1371/journal.pone.0035598 22530057 PMC3329458

[6] Brouat C, Rahelinirina S, Loiseau A, Rahalison L, Rajerison M, Laffly D, et al (2013) Plague Circulation and Population Genetics of the Reservoir *Rattus rattus*: The Influence of Topographic Relief on the Distribution of the Disease within the Madagascan Focus. PLoS Negl Trop Dis 7(6): e2266 10.1371/journal.pntd.0002266 23755317 PMC3674990

[7] National Plague Control Guidelines. Department of Health, Republic of South Africa. (available at https://www.nicd.ac.za/assets/files/National_Plague_Control_Guidelines.pdf)

[8] Ziwa MH, Matee MI, Hang’ombe BM, Lyamuya EF, and Kilonzo BS. (2013). Plague in Tanzania: An overview. Tanzania Journal of Health Research 15(4), 10.4314/thrb.v15i4.7 26591701

